# Identifying sensors-based parameters associated with fall risk in community-dwelling older adults: an investigation and interpretation of discriminatory parameters

**DOI:** 10.1186/s12877-024-04723-w

**Published:** 2024-02-01

**Authors:** Xuan Wang, Junjie Cao, Qizheng Zhao, Manting Chen, Jiajia Luo, Hailiang Wang, Lisha Yu, Kwok-Leung Tsui, Yang Zhao

**Affiliations:** 1 Intelligent Sensing and Proactive Health Research Center, School of Public Health (Shenzhen), Sun Yat-sen University, Shenzhen, China; 2 School of Design, the Hong Kong Polytechnic University, Hung Hom, Hong Kong; 3 Grado Department of Industrial and Systems Engineering, Virginia Polytechnic Institute and State University, Blacksburg, VA USA

**Keywords:** Fall risk, Timed up and go test, Older adults, Inertial measurement unit, Depth camera

## Abstract

**Background:**

Falls pose a severe threat to the health of older adults worldwide. Determining gait and kinematic parameters that are related to an increased risk of falls is essential for developing effective intervention and fall prevention strategies. This study aimed to investigate the discriminatory parameter, which lay an important basis for developing effective clinical screening tools for identifying high-fall-risk older adults.

**Methods:**

Forty-one individuals aged 65 years and above living in the community participated in this study. The older adults were classified as high-fall-risk and low-fall-risk individuals based on their BBS scores. The participants wore an inertial measurement unit (IMU) while conducting the Timed Up and Go (TUG) test. Simultaneously, a depth camera acquired images of the participants’ movements during the experiment. After segmenting the data according to subtasks, 142 parameters were extracted from the sensor-based data. A t-test or Mann-Whitney U test was performed on the parameters for distinguishing older adults at high risk of falling. The logistic regression was used to further quantify the role of different parameters in identifying high-fall-risk individuals. Furthermore, we conducted an ablation experiment to explore the complementary information offered by the two sensors.

**Results:**

Fifteen participants were defined as high-fall-risk individuals, while twenty-six were defined as low-fall-risk individuals. 17 parameters were tested for significance with *p*-values less than 0.05. Some of these parameters, such as the usage of walking assistance, maximum angular velocity around the yaw axis during turn-to-sit, and step length, exhibit the greatest discriminatory abilities in identifying high-fall-risk individuals. Additionally, combining features from both devices for fall risk assessment resulted in a higher AUC of 0.882 compared to using each device separately.

**Conclusions:**

Utilizing different types of sensors can offer more comprehensive information. Interpreting parameters to physiology provides deeper insights into the identification of high-fall-risk individuals. High-fall-risk individuals typically exhibited a cautious gait, such as larger step width and shorter step length during walking. Besides, we identified some abnormal gait patterns of high-fall-risk individuals compared to low-fall-risk individuals, such as less knee flexion and a tendency to tilt the pelvis forward during turning.

**Supplementary Information:**

The online version contains supplementary material available at 10.1186/s12877-024-04723-w.

## Introduction

Falls have been recognized as a major health problem for older adults with significant physical and psychological consequences [1]. Community-dwelling older adults (age ≥ 65 years) experience 0.3–1.6 falls per person annually, of which approximately 5% result in fractures or require hospitalization [2]. As reported [3], falls have become the second leading cause of injury-related deaths among older adults worldwide, and they are a major cause of both death and injury in people over 65 years of age. Existing literature shows that several inherent risk factors can contribute to different gait and balance deficits that would result in falling, such as decreased muscle strength, limited range of movement, abnormal muscle tone, lack of motor coordination, poor sensory organization, and cognitive deficit [4]. It is crucial to reduce the burden of the disease brought by falls. Studies have shown that timely identifying people at risk of falls and implementing fall prevention programs can successfully reduce fall occurrences [5].

There are some clinical measurements that were widely used to evaluate the fall risk of older adults, such as the Berg Balance Scale (BBS), Tinetti Performance Oriented Mobility Assessment (POMA), Balance Evaluation System Test (BESTest), and Functional Gait Assessment (FGA) [6–8]. Among these BBS is a 14-item scale that demonstrates a strong correlation with prospective fall events [9]. It has been widely employed in the assessment of fall risk and has proven its effectiveness in various research investigations [10, 11]. However, it takes 15–20 min and requires expert supervision to perform the BBS test [10]. Conventional assessment instruments entail considerable time and effort, which impedes their rapid and extensive application at the communal level [12].

The advancement of information technology and sensor-based methodologies has facilitated the implementation of efficacious fall risk assessment techniques. Sensors, through the extraction of objective and quantifiable human motion data, have been validated in multiple studies for their effectiveness in fall risk assessment [13–16]. These techniques contain sophisticated information, provide comprehensive data, and can significantly reduce the time and labour required for assessment. Among these methods, nearable (environmental perception) and wearable sensors are widely employed due to their high efficacy. Typical examples of environmental perception sensors are infrared sensors, Doppler radar, depth camera systems, and force platforms [17–20] which facilitate accurate and precise monitoring, detection, and analysis of various environmental parameters. Typical examples of wearable sensors are pressure insoles and inertial measurement units (IMUs) [21, 22] which can monitor human activities in real-time and provide kinematic data for subsequent analysis.

The integration of standard tests and sensor-based devices can not only simplify the fall risk assessment process, but also assess fall risk in older adults accurately by extracting gait parameters during their movement [23, 24]. Previous studies have demonstrated the validity and reliability of each type of sensor individually [16, 25–27]. Due to variations in measured parameters and modeling techniques, reported accuracy ranges of fall risk assessments in different studies vary widely, ranging from 47.9 to 100% [14]. There was various existing research concerning gait parameters extracted from IMU or depth camera data of the Timed Up and Go (TUG) test assessing mobility or balance among older adults. Diao et al. conducted an IMU-instrumented, environment-adapting TUG test to extract and quantify gait parameters potentially associated with falls. The results indicate that parameters extracted by wearable inertial sensors could effectively differentiate individuals with high and low fall risks [15]. Dubois et al. automated the TUG test with the Microsoft Kinect v2 application. Based on the Kinect data collected from tests, several parameters relating to the gait and turn pattern, the sitting position, and the duration of each phase were extracted to build an evaluation system for the mobility and balance of individuals. The evaluation system can objectively quantify the change in parameters of different subtasks of TUG to provide discrimination of high risk for falls in older adults [28].

The combination of IMUs and depth cameras for gait and fall risk analysis is a novel and promising approach that has received growing attention in the literature. In the realm of utilizing sensors for fall risk assessment, a prevalent approach involves the use of a single type of sensor [14, 22, 29]. However, the simultaneous use of nearable and wearable sensors is only observed in a subset of fall monitoring studies [30, 31]. This research gap motivates further exploration of the potential advantages of integrating these two types of sensors in fall risk assessment. While IMUs can precisely record the movement of particular body parts, more IMUs are necessary to record additional parts. Conversely, depth camera can capture more key points during motion despite their lack of precision. Their complementary and synergistic effects have not been thoroughly examined. Therefore, we combined parameters from the IMU and depth camera used in previous studies to investigate their role in fall risk assessment among community-dwelling older adults. From a clinical perspective, employing sensors for fall risk assessment can alleviate the burden on healthcare professionals and facilitate large-scale, long-term monitoring. Our emphasis is on interpreting the meaning of the parameters, with the goal of providing additional clinical insights into individuals at high risk.

This study utilized depth camera technology and IMU to measure gait parameters in geriatric community members. The objective was to identify factors distinguishing between individuals at high and low risk of falls, utilizing 142 parameters primarily derived from the 3 m-TUG subtask. These parameters encompassed demographic, gait, kinematic, and anatomical variables. Logistic regression models were employed to evaluate the contribution of these factors to fall risk. The paper aimed at discussing and interpreting the significant parameters associated with fall risk. This discussion considered the physiology of older adults and drew insights from relevant literature. Furthermore, it delineated the potential complementarity of depth camera technology and IMUs for gait assessment.

## Methods

### Participants

We recruited older adults who met all the following inclusion criteria: at least 65 years old, living in a community setting, having the ability to walk independently or with walking assistance, with normal (or corrected-to-normal) vision, and having the ability to provide informed consent. We excluded older adults with abnormal vision, physical impairments, and life-threatening illnesses, as they were likely unable to complete the gait and balance assessment. The pilot study was approved by the Research Ethics Committee of the City University of Hong Kong (reference number: 3-2020-02-F). All participants provided written informed consent before the initiation of our study.

The data collection was conducted between December 2019 and January 2020, and fifty-four participants from 4 different community settings were recruited based on the inclusion and exclusion criteria. However, only forty-one participants (thirty-six women and five men) aged 68–89 years (79.07 ± 6.47 years) completed all the tests due to some participants declining the depth camera recording or withdrawing from the experiment. These community settings are distributed across different districts in Hong Kong, without fixed residential types, thereby endowing the results with a high degree of generalizability.

### Assessment and protocol

During the experiment phase, participants were required to complete two sets of tests: the BBS assessment and the 3 m-TUG test.

**Fig. 1:**
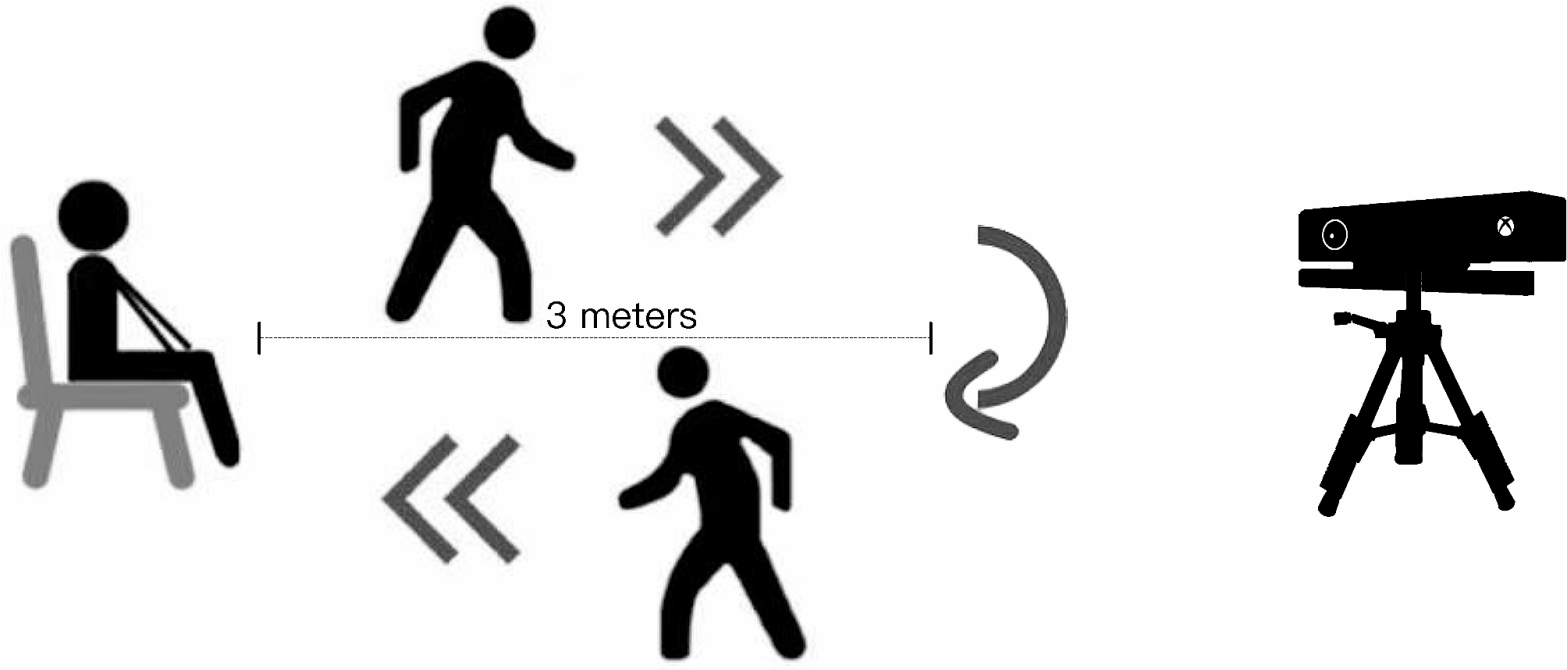
The process of 3 m-TUG.

- *BBS*: The BBS consists of 14 items examining the balance capability of older adults, and each item is scored on a scale of 0 to 4. If the participant is unable to do the task, the score is recorded as 0, and if the participant is able to complete the task according to the criteria assigned to it, the score is 4. The maximum total score on the test is 56. The BBS was developed as a balance assessment to objectively assess a person’s ability to control balance in a functional task [32]. For the prediction of falls in community-dwelling older adults, a cutoff score of 50 was proposed [33]. Participants were labeled as low-fall-risk individuals if their BBS score was > 50. Otherwise, they were labeled as high-fall-risk individuals. Therefore, we identified twenty-six low-fall-risk individuals and fifteen high-fall-risk individuals.
- *3 m-TUG test*: participants were required to stand up from a chair, walk 3 m, turn around 180°, then walk back around and sit in the chair again [34]. The TUG is a simple and feasible tool that can be administered in any setting and at any time [35]. It involves basic mobility skills such as rising, turning, sitting, and walking. Therefore, it is recommended as a screening tool to identify older adults with balance or gait impairments [36]. Figure 1 demonstrates the general process of 3 m-TUG. In our experiment, all participants finish the TUG without any walking assistance.

During the experiment, we used a commercial IMU sensor (9 Degrees of Freedom) and a Kinect (Microsoft Kinect for Windows v2) device to capture the participants’ movement parameters while they completed the 3 m-TUG.

**Fig. 2:**
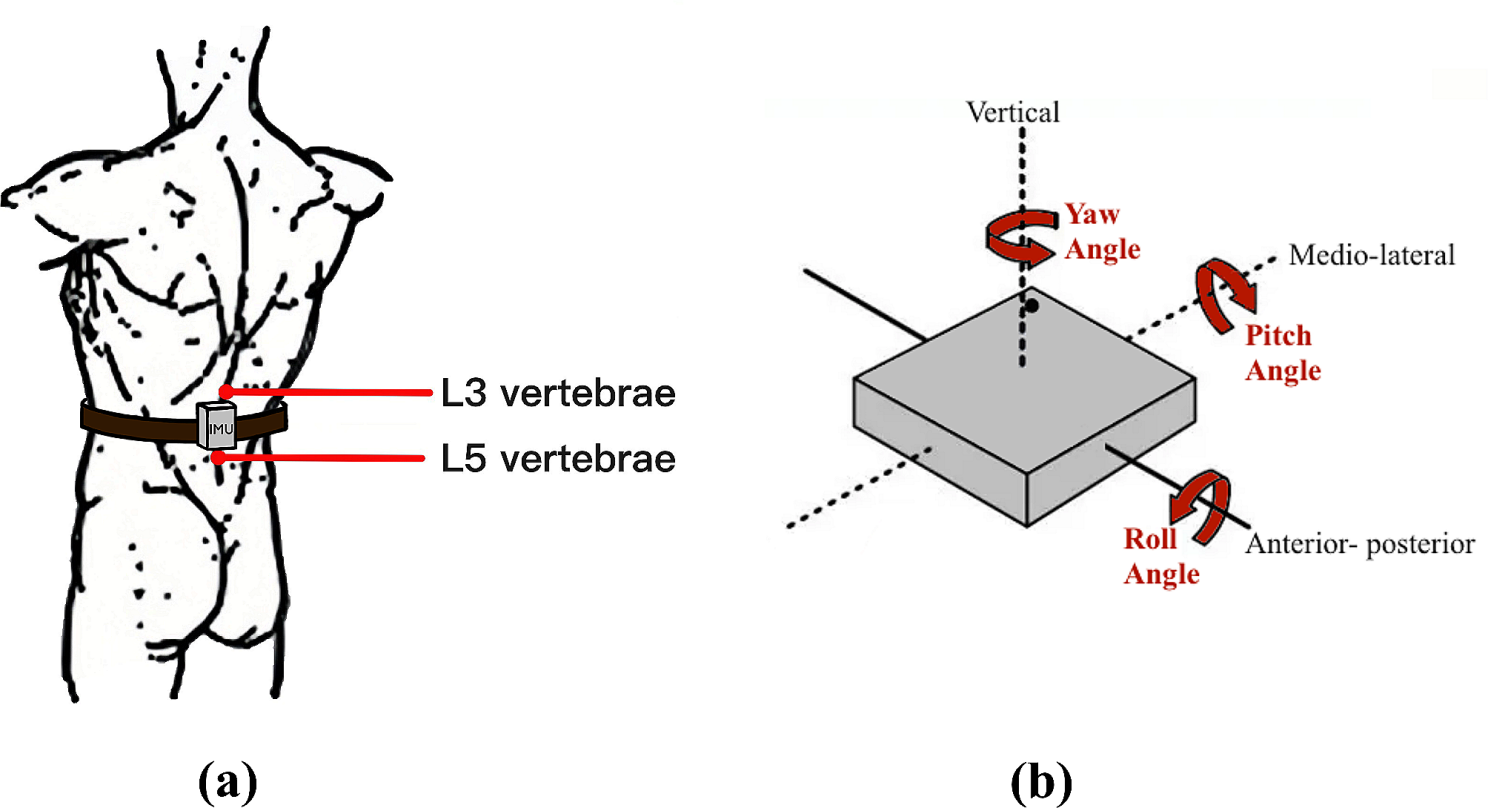
(**a**) The IMU is positioned between L3 and L5 vertebrae. (**b**) IMU’s three axes

**Fig. 3:**
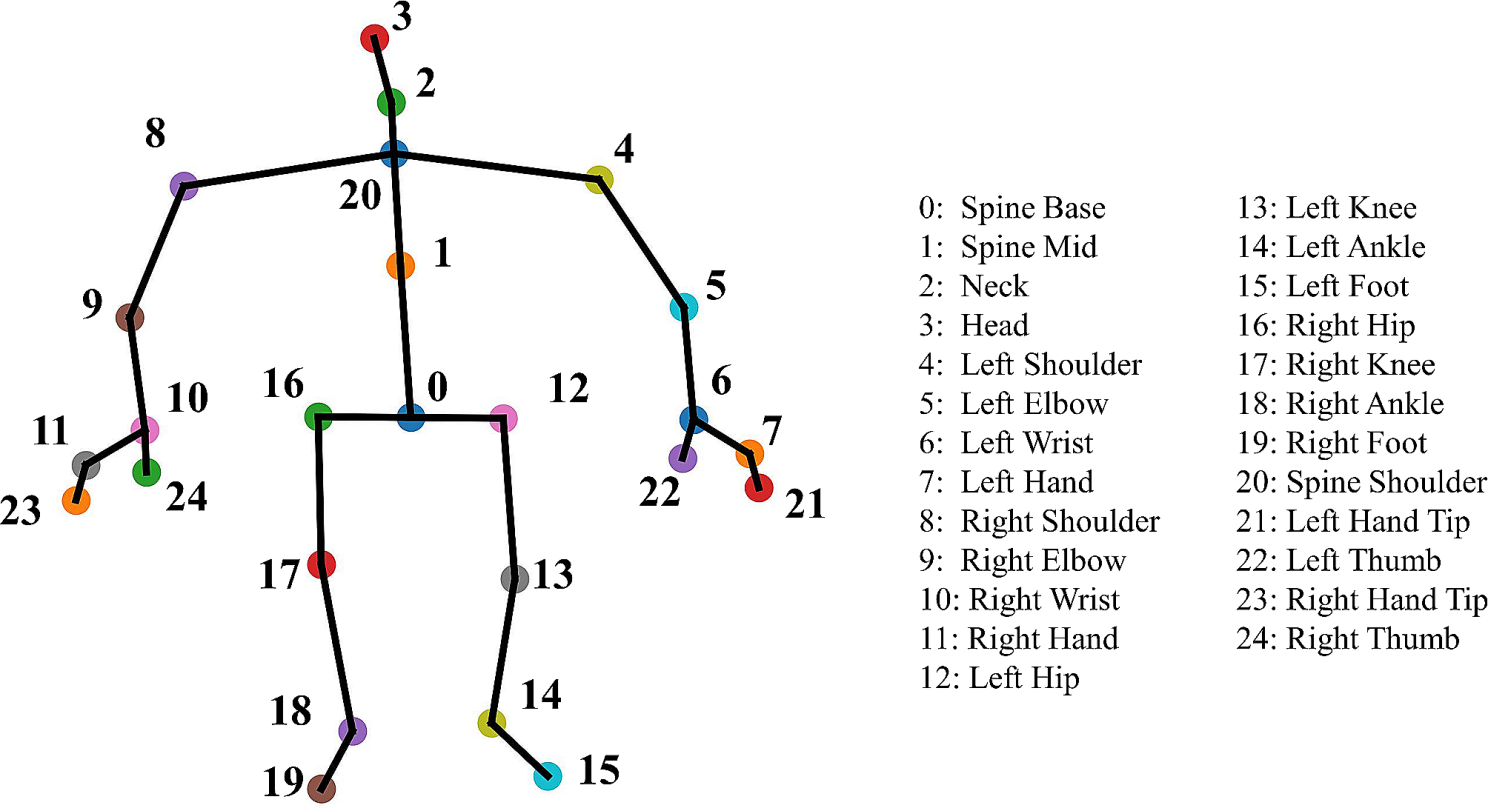
25 human key-point detected by Kinect

- *IMUs* are widely used for the analysis of gait parameters and fall risk assessment due to their accuracy, portability, and low price [37]. IMU with a sampling frequency of 5 Hz consisting mainly of an accelerometer and a gyroscope was placed on the participants’ lower back area between the L3 and L5 vertebrae. Acceleration signals were derived from three axes: vertical (V), mediolateral (ML), and anterior-posterior (AP). Angular velocity of a gyroscope: yaw, pitch, and roll rotate around V, ML, and AP, respectively, as shown in Fig. 2.
- *Kinect* is a portable device with an integrated depth sensor and a micro camera. Different from the traditional camera system, the depth camera technology obtains 3D information about subjects with the application of the stereo calibration method, which can produce RGB images and dynamically capture the movement of the human body for accurate analysis of human posture [38, 39]. As Fig. 3 shows, the key-point data collected from Kinect is presented in the form of 25 human skeletal joints in the three-dimensional coordinate, which means that the motion of each key-point can be studied individually.

### Divide the TUG into subtasks

**Fig. 4:**
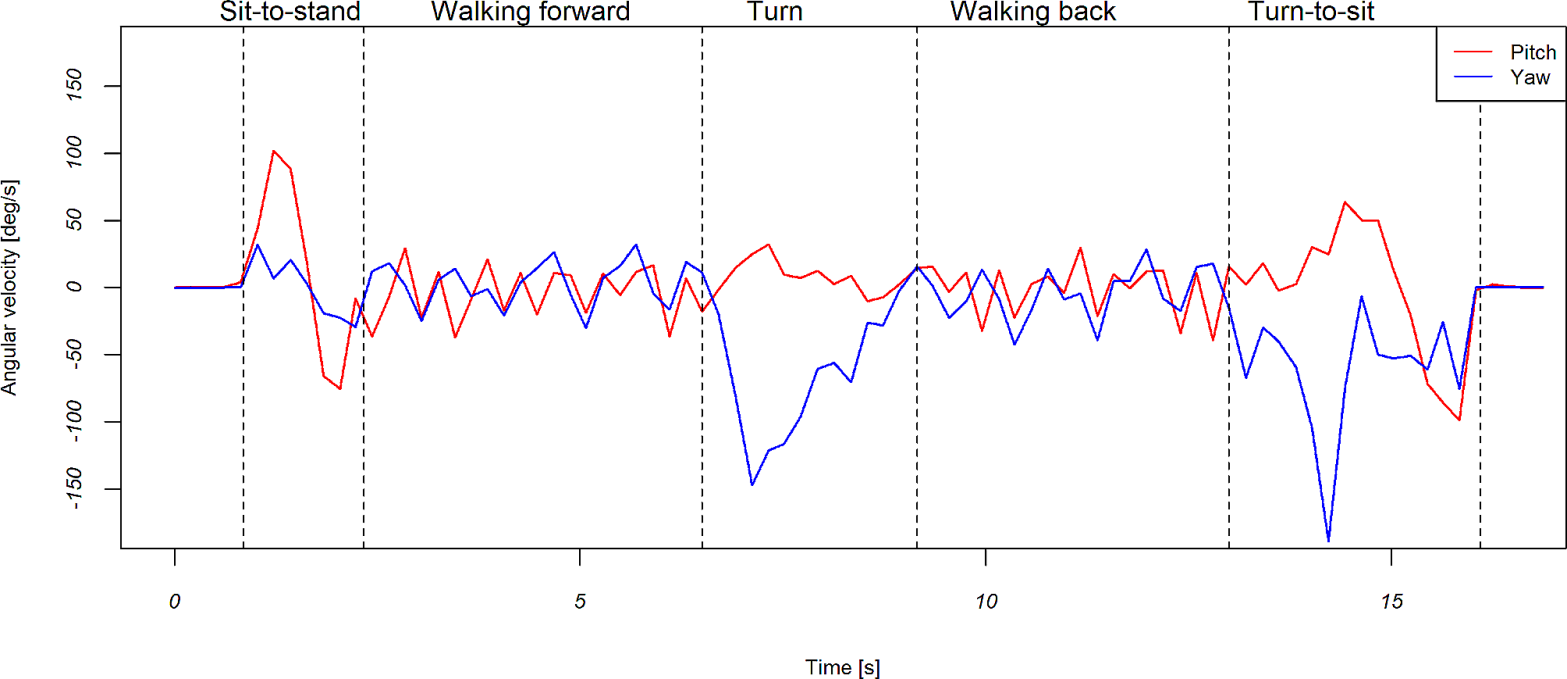
An example of the gyroscope data of a person during the TUG test

Dividing the TUG into subtasks can provide a more detailed and nuanced assessment of mobility and functional ability in older adults. Figure 4 shows an illustrated example of the gyroscope data of a person during the TUG test. TUG was divided into five subtasks: sit-to-stand, walking forward, turn, walking back, and turn-to-sit. The five segmentations were detected with angular velocities in the yaw and pitch axes using the developed algorithm presented by previous related work [40, 41]. Data segmentation was achieved by capturing transitions in axis fluctuations. The algorithm leverages stable angular velocity values during sitting, facilitating the identification of TUG test initiation and termination. The pitch axis experiences significant fluctuations in sit-to-stand and stand-to-sit phases, while the yaw axis exhibits pronounced peaks during turning. Notably, both pitch and yaw show minimal fluctuations during the walking phase.

### Parameters extraction and statistical analysis

The goal of this analysis was to identify the significant difference between high-fall-risk individuals and low-fall-risk individuals. Each subtask provides different physical function information, so the parameters were extracted based on different tasks. A walking phase is created by merging the two walking segments. The study examined a total of 142 parameters that have been employed in previous studies on fall risk identification [16, 25–27]. Table 1 shows the main parameters extracted from IMU and Kinect.

- 8 parameters were obtained from the subtasks’ temporal data: duration is the time required to complete the subtasks; the percentage of duration (POD) is the subtask duration divided by the TUG duration.
- 3*22 parameters were obtained from acceleration data: the maximum, minimum, median, range, root mean square (RMS), coefficient of variation (CV), maximum jerk (Max jerk), and mean jerk of the triaxial accelerometers during each subtask were calculated. It is noteworthy that all parameters were computed for the entire subtask. For example, in the Sit-to-stand task, the Max jerk corresponds to the maximum change in acceleration during this specific task period [16, 42], calculated as the maximum difference in acceleration values.
- 3*18 parameters were obtained from angular velocity data: the maximum, minimum, median, range, RMS, and CV of the triaxial angular velocity during each subtask were calculated.
- 10 parameters were obtained from key-point data: legs angle is the angle between the two legs, defined as the angle between the two vectors of the line between the joints 0 and 17 and the line between 0 and 13 (Fig. 3); knee angle is the angle between the thigh and the calf, for example, the right knee angle is the angle between the two vectors 17 and 16 of the joint and 17 and 18 of the joint (Fig. 3); step counts is the number of steps during walking; step length is the total length of walking (6 m) divided by the number of steps; step duration is the duration of walking divided by the number of steps; step width is the average width between the two feet.
- 1 parameter was the duration of the TUG test.
- 3 parameters were obtained from demographic information, including gender, age, and the use of walking assistance.

These parameters can be classified into four categories based on previous studies [26, 43], the first category of parameters being demographic parameters (e.g., age); the second category being kinematic parameters extracted from the IMU (e.g., maximum acceleration, the angular velocity at turn); the third category being gait parameters (e.g., step count, step length, and walking duration); and the fourth category being anatomical parameters (e.g., knee angle). For all extracted parameters, after checking the normality of the distribution within the two groups using the Shapiro-Wilk test, statistical comparisons between high-fall-risk individuals and low-fall-risk individuals were conducted. The tests employed included the t-test or Mann-Whitney U test, with a significance level of$\alpha =0.05$. The Cohen’s *d* parameter was calculated to obtain the effect size for each variable, aiming to assess its discriminative power [10]. The value of *d* greater than 0.8 is considered to have a large effect size [44]. Univariate logistic regression was employed to assess parameters with a large effect size. This approach allowed us to further quantify the role of different parameters in identifying individuals at high risk of falls. Additionally, we conducted ablation experiments using these parameters in conjunction with multivariate logistic regression to examine whether the information from the two sensors is complementary. Statistical analyses were conducted using the R-4.2.1.

**Table 1:** Summary of the main parameters extracted from IMU and Kinect

	Sit-to-stand	Walking	Turn	Turn-to-sit
Temporal(8)	Duration(G), POD(G)	Duration(G), POD(G)	Duration(G), POD(G)	Duration(G), POD(G)
Acceleration(3*22) (V, ML, AP)	Maximum(K), Minimum(K), Range(K), RMS(K), Max jerk(K), Mean jerk(K)	Range(K), RMS(K), Median(K), CV(K)	Range(K), RMS(K), Median(K), CV(K)	Maximum(K), Minimum(K), Range(K), RMS(K), Median(K), CV(K), Max jerk(K), Mean jerk(K)
Angular velocity(3*18) (yaw, pitch, roll)	Maximum(K), Minimum(K), Range(K), RMS(K)	Range(K), RMS(K), Median(K), CV(K)	Range(K), RMS(K), Median(K), CV(K)	Maximum(K), Minimum(K), Range(K), RMS(K), Median(K), CV(K)
Key-point(10)		Legs angle(A), Left knee angle(A), Right knee angle(A), Step counts(G), Step length(G), Step duration(G), Step width(G)	Legs angle(A), Left knee angle(A), Right knee angle(A)	

*Note*: Each parameter is annotated with its type: K for kinematic parameters, D for demographic parameters, G for gait parameters, and A for anatomical parameters. In addition to the 138 parameters presented in this table, there is an extra parameter indicating the duration of the TUG test, along with three demographic parameters

## Results

### Demographic parameters of participants

The general parameters of the participants are presented in Table 2. It can be seen that the two groups differ significantly in the parameters ‘Age’ and ‘usage of walking assistance’ in daily life. Compared with low-fall-risk individuals, older age and use of walking assistance in daily life were observed in high-fall-risk individuals, as expected.

**Table 2:** Demographic parameters of the participants

Parameter	Low-fall-risk individuals	High-fall-risk individuals	p-Values
Participants (n)	26	15	–
Age (years ± standard deviation)	77.38 ± 5.34	82.00 ± 7.36	0.041
Gender (M/F)	2/24	3/12	0.262
Usage of walking assistance (Yes/NO)	4/22	10/5	0.001

### Significant parameters extracted from IMU and Kinect

Seventeen parameters showed significant differences between the two groups. Table 3 shows the *p*-values and effect size of the significant parameters. The utilization of walking aids, Max-Ang-Yaw during the turn-to-sit phase, and step length exhibited the most substantial effect sizes in discriminating between the two groups. The low-fall-risk group demonstrated reduced reliance on walking aids in their daily activities. Notably, during the turn-to-sit phase, the angular velocity in yaw manifested a higher maximum value, and the step length during walking was greater for low-fall-risk individuals. Complete results, including test results for 142 parameters, are provided in the Additional file 1.

**Table 3:** Significant parameters (*p* < 0.05)

Parameter	Subtask	Axis	Low-fall-risk individuals	High-fall-risk individuals	p-Values	Effect size
CV [deg/s]	Turn	Pitch	2.19 ± 8.77	-3.75 ± 6.31	< 0.001	0.745
Usage of walking assistance [Yes/NO]	–	–	4/22	10/15	0.001	1.236
Max [deg/s]	Turn-to-sit	Yaw	161.28 ± 44.40	122.25 ± 33.24	0.003	0.957
CV [deg/s]	Turn-to-sit	Yaw	1.31 ± 0.31	1.09 ± 0.15	0.005	0.814
Step length [m]	Walk	–	0.37 ± 0.07	0.31 ± 0.06	0.007	0.909
Step width [m]	Walk	–	0.13 ± 0.03	0.16 ± 0.03	0.008	0.859
Median [deg/s]	Turn	Pitch	2.10 ± 6.45	-2.80 ± 5.86	0.021	0.786
Right knee angle [deg]	Walk	–	158.95 ± 5.48	162.18 ± 3.19	0.022	0.673
RMS [deg/s]	Walk	Pitch	8.16 ± 2.41	6.64 ± 1.77	0.026	0.692
Max [m/s^2^]	Turn-to-sit	V	1.14 ± 0.05	1.19 ± 0.07	0.026	0.859
Mean jerk [m/s^2^]	Turn-to-sit	AP	0.10 ± 0.03	0.08 ± 0.02	0.029	0.641
POD [%]	Walk	–	39.27 ± 6.53	43.43 ± 6.74	0.031	0.624
Right knee angle [deg]	Turn	–	157.71 ± 10.60	164.44 ± 9.05	0.034	0.666
Age [years]	–	–	77.38 ± 5.34	82.00 ± 7.36	0.041	0.752
Mean jerk [m/s^2^]	Sit-to-stand	ML	0.04 ± 0.02	0.05 ± 0.02	0.050*	0.456
RMS [deg/s]	Turn-to-sit	Yaw	71.24 ± 24.6	57.22 ± 17.33	0.050*	0.630
Max [deg/s]	Turn-to-sit	Roll	18.72 ± 11.3	26.67 ± 11.55	0.050*	0.698

*Note*: The results of ranking for 17 significant parameters’ *p*-values from smallest to largest for the t-test or Mann-Whitney U test. The values on each parameter were reported as mean ± standard deviation. The asterisk (*) denotes that the displayed value is 0.050, constrained by significant figures. When retaining six significant figures, all three *p*-values are 0.049724. The effect size is represented by Cohen’s *d* value

### Logistic regression model

We employed univariate logistic regression to investigate the association between parameters demonstrating significance and a large effect size (*d* > 0.8) with the risk of falls. The parameters considered included the usage of walking assistance, Max-Ang-Yaw during turn-to-sit, CV-Ang-Yaw during turn-to-sit, step length, step width, and Max-Acc-V during turn-to-sit, as presented in Table 4.

**Table 4:** Univariable logistic regression of fall risk

Parameter	Subtask	Axis	B	p-Values	OR	95% CI
assistance	–	–	2.398	0.002	11.000	(2.619,56.297)
Max	Turn-to-sit	Yaw	-0.026	0.013	0.974	(0.951,0.992)
CV	Turn-to-sit	Yaw	-3.767	0.027	0.023	(0.001,0.44)
Step length	Walk	–	-13.429	0.015	0.000	(0.000,0.032)
Step width	Walk	–	30.019	0.021	1.090*10^13^	(1042.921,5.385*10^25^)
Max	Turn-to-sit	V	14.316	0.020	1.649*10^6^	(22.283,1.053*10^12^)

The findings suggest that heightened utilization of walking assistance, reduced variability in Yaw during the turn-to-sit subtask, and diminished step length during walking are associated with an increased risk of falls. Although all participants were able to perform the TUG without walking aids in our experiments, the historical use of such aids in their daily lives still plays a key role in assessing fall risk. Conversely, an expanded step width and elevated maximum velocity during the Turn-to-sit subtask are indicative of significantly heightened fall risk.

**Fig. 5:**
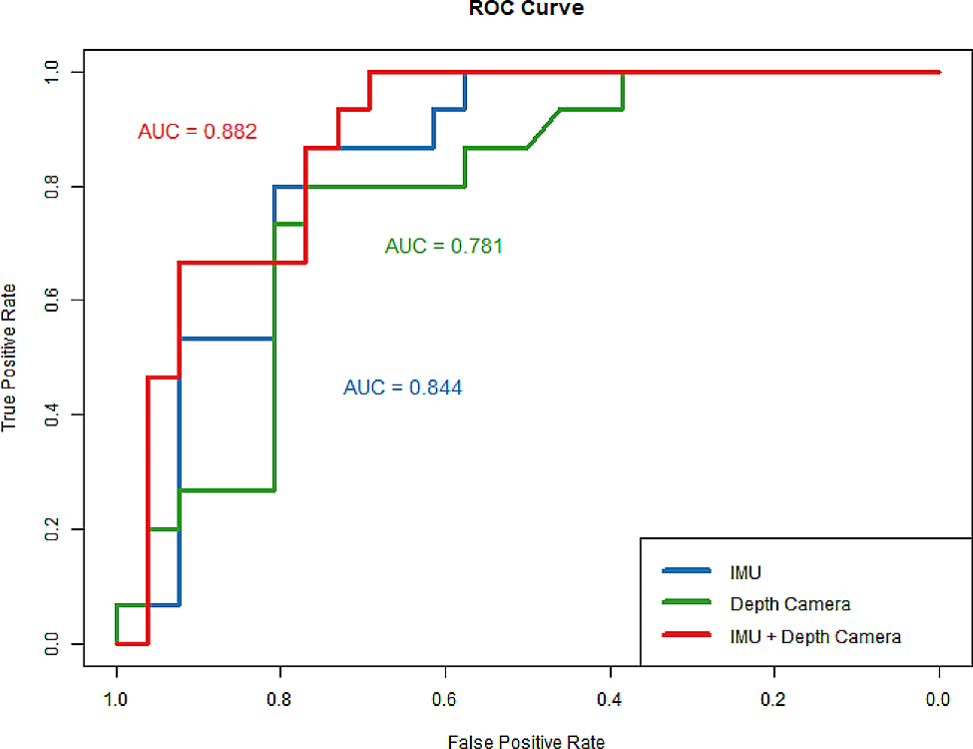
ROC curve of different feature subsets

To assess whether different sensor-extracted features contribute complementarily to the assessment of fall risk, we conducted an ablation experiment. Three feature sets were employed (IMU feature set: Max-Ang-Yaw during turn-to-sit, CV-Ang-Yaw during turn-to-sit, and Max-Acc-V during turn-to-sit; Depth Camera feature set: step length, step width; combination of IMU and Depth Camera) to construct multivariate logistic regression models. The area under the curve (AUC) values were computed. The results, as depicted in Fig. 5, indicate that the combined use of IMU and Depth Camera features enhances the classification AUC values (AUC = 0.882).

## Discussion

### General considerations

An important contribution of this study is the quantitative assessment of multiple parameters associated with fall risks among community-dwelling residents aged 65 years old and above. Our results indicate that 17 parameters are relevant for assessing the fall risk of older adults, comprising 11 kinematic parameters, 3 gait parameters, 2 demographic parameters, and 1 anatomical parameter. This suggests that electronic devices such as IMUs and Kinect can extract relevant features to facilitate the detection of gait abnormalities in older adults, reducing the reliance on healthcare professionals for manual assessment of fall risk.

Participants performed a simple TUG test, which is a widely used assessment tool for older adults [45]. Various subtasks within the test can be employed to assess different aspects of daily living activities in the elderly. Sit-to-stand involved lifting from a chair with momentum transfer and body stabilization [46]. Walking required regulating angular momentum for fall prevention, employing “elevating” and “lowering” strategies [47]. Turning involved translating and rotating the body while maintaining dynamic stability [48]. Stand-to-sit required synchronized trunk and knee control for center of gravity displacement [49]. The choice of the TUG aligns with our objective to efficiently gather rich and diverse data on balance abilities in a real-world context, facilitating a nuanced exploration of balance-related parameters across different TUG subtasks. We identified more significant parameters in the turn-to-sit phase compared to the other phases, with seven in total. This is followed by five significant parameters in the walking phase, where previous studies have demonstrated the highest correlation between walking and the incidence of falls [50].

### Interpretation of parameters associated with fall risk

Using TUG duration alone to distinguish fallers from non-fallers has long been controversial. Although similar to the results of previous studies [6], the TUG duration in this experiment was longer in high-fall-risk individuals than in low-fall-risk individuals, but the *p*-value was nonsignificant (high-fall-risk individual: 16.11 ± 6.36, low-fall-risk individual: 13.53 ± 3.52, *p* = 0.208). A systematic review [51] noted that the TUG duration is not useful in distinguishing fallers from non-fallers in a healthy state but is more valuable in less-healthy older adults. This is consistent with our results because our participants were older healthy residents from the community, so no significant TUG time threshold was found to identify high risk in the older adults. However, we found that the percentage of the duration (POD) of walking is a significant parameter to distinguish between the two groups (high-fall-risk individual: 43.43 ± 6.74, low-fall-risk individual: 39.27 ± 6.53, *p* = 0.031). Motor control is a complex interplay of cognitive and sensorimotor systems, and studies have demonstrated that the speed of information processing declines with age (i.e., time increases) by approximately 26% [52]. The difference in the POD of walking between the two groups may be associated with the different speeds of information processing for postural transitions or different walking strategies.

We also found that the demographic parameters were meaningful in distinguishing the two populations. Older adults or those who use walking assistance are more likely to be defined as high-fall-risk individuals. This result is consistent with the reality that the rate of falls among older adults increases with age [53, 54]. A correlation between age and BBS score was also mentioned in a study evaluating age and gender-related balance performance in older adults [55]. The TUG is a brief activity that all participants were able to complete without aids. However, some individuals may require assistive devices for more frequent or prolonged daily activities to maintain balance. The reliance on assistive devices in the elderly’s daily life suggests potential issues with their balance or gait abilities. Another study [56] assessing the risk of falls among nursing home residents showed that although more people in the falls population used walking assistance, the *p*-value comparing fallers and non-fallers using whether or not walking assistance was nonsignificant. One potential reason for the inconsistency between this result and ours is that our subjects were older adults generally in good physical condition living in the community, with 34% of them using walking assistance. In contrast, in this study, it was nursing home residents with 49% walking assistance use.

Older adults are more likely to be perceived as walking carefully, hobbling, and having short strides [57]. This walking characteristic is usually considered a sign of fear of falling in older people, which aligns with the “cautious gait” concept introduced by physiologists. The introduction of “cautious gait” [58] explains many of the movement patterns of older adults. The results of our experiments show that high-fall-risk individuals have a more cautious gait, a greater step width and a smaller step length to maintain body balance. There is also some evidence for a careful walking strategy in older adults, such as smaller values of CV, Rms, etc., for the angular velocity of yaw rotation in the turn-to-sit phase in high-fall-risk individuals than in low-fall-risk individuals. Many older adults exhibit a timid, reserved, and slow gait as a consequence of the fear of falling [59]. This fear can have detrimental effects on the well-being of older adults, such as social withdrawal, depressive symptoms, and elevated fall risk [60].

Dierick et al. [56] conducted a fall risk assessment in nursing homes using the TUG test. They found that non-fallers exhibited a higher Max-Ang-Yaw than fallers during the first turn, suggesting that older adults with a higher fall risk had a lower turning velocity. Our results showed that a similar phenomenon was found during the turn-to-sit phase (high-risk individual: 122.25 ± 33.24, low-risk individual: 161.28 ± 44.40, *p* = 0.003). The second turn involves more cognitive skills than the first turn, as it requires planning the process of sitting down [61]. Older adults with turn difficulties adopted multi-step turns as a compensatory strategy to reduce the complexity and coordination demands of pivoting turns, which were preferred by younger and older adults without turn difficulties [62]. This strategy resulted in a significantly lower Max-Ang-Yaw among high-fall-risk individuals. Multi-step turns involve fewer feedback mechanisms and allow for smaller or slower movements that are easier to execute.

Also, the significance test results showed that the CV-Ang-Pitch during the turn was the most significant parameter in distinguishing between the two populations, and Median-Ang-Pitch during the turn was also significant. Both parameters indicated a tendency to lean forward in the position where the IMU of the high-fall-risk individuals was located during the turn. This is related to the presence of anterior pelvic tilt in the gait of older adults [63]. A study of joint angles in older and younger people showed that older subjects had more anterior pelvic tilt during walking than younger people [64]. In older adults, anterior pelvic tilt may be caused by postural changes caused by weakness in the abdominal and hip muscles or as compensation for fear of instability [65].

Another interesting phenomenon is that high-fall-risk individuals have a greater right keen angle during the walking and turning phases, i.e., less knee flexion. A study [64] showed that knee range of motion was also reduced in older adults (55 ± 5°) compared to younger adults (59 ± 5°) during the gait cycle. Increased knee flexion during walking can produce greater eccentric work [66]. A lower knee flexion angle may indicate impaired eccentric control of the knee, resulting in more difficult balance control and greater susceptibility to falls [67].

We recommend utilizing parameters with large effect sizes for analyzing the fall risk in older adults. Among all parameters extracted by IMU, the maximum angular velocity around the yaw axis during turn-to-sit exhibits optimal discriminatory power (i.e., the largest effect size) in distinguishing between high and low fall risks. According to the logistic regression results, during the Turn-to-sit subtask, a one-unit decrease in the maximum yaw angle is associated with a 2.6% reduction in the odds of fall risk. This result provides new evidence supporting the previous conclusion that turning speed induces changes in overall coordination among older adults [68]. Among all parameters measured by the depth camera, step length exhibits the highest discriminatory power and is considered a protective factor. Slower gait and shorter step length in high-fall-risk individuals are considered to enhance stability against balance threats [69].

### Complementary effects of IMU and depth camera

IMU and depth camera technologies have been widely validated for assessing fall risk in the elderly [28, 70, 71]. They are considered low-cost and user-friendly tools for human motion analysis. However, their complementary roles are rarely addressed in the literature. Both theoretically and practically, it seems evident that these two sensors provide a certain degree of complementarity. IMU, as a prevalent wearable sensor, is extensively utilized in research [37]. It offers detailed acceleration and angular velocity data, reflecting nuanced movement patterns [72]. However, its applicability is limited to specific wearing locations, and the optimal placement has been a subject of research discussion [73].On the other hand, depth cameras employ built-in and externally validated skeletal modeling algorithms to provide markerless 3D motion tracking of multiple joints in the body [14]. Nevertheless, they face challenges in offering detailed motion information, such as angular velocity. We extracted various kinematic parameters from IMU signals and multiple gait and anatomical parameters from the depth camera, some of which are device-specific, like knee angle, which can only be obtained from the depth camera. To understand their roles in fall risk assessment, we conducted ablation experiments using logistic regression. The results indicated that combining features from both sensors leads to a higher AUC value. However, we acknowledge that this is a preliminary validation, and further evidence is necessary to substantiate the complementary nature of the two devices.

### Limitations and future work

This study has several limitations. The sampling frequency of IMU is only 5hz, which may result in a partial loss of information. A greater percentage of elderly females than males took part in the experiment in the community, which is connected to the fact that elderly females are more engaged in health-related activities [74]. The systematic imbalance in demographic parameters and small sample size may limit the generalizability of the results. The limited adoption of depth cameras among the elderly population is another issue that merits consideration. The utilization of a clinical assessment to infer fall risk, rather than prospectively monitoring fall occurrences, introduces an additional limitation to the study.

The 5 Hz sampling frequency constitutes a significant limitation in our study, requiring subsequent exploration of the consistency between low-frequency and high-frequency sampling information. Investigating the variability between individuals (i.e., differences in completing the TUG task each time) and its potential impact on assessment results will be a direction for future research. Additionally, we are interested in exploring the potential role of significant parameters in predicting high-fall-risk individuals in real-world settings. To this end, we will use interpretable machine learning algorithms to verify these parameters’ impact on prediction accuracy and gain further insight into the underlying mechanisms behind fall risk.

## Conclusion

The high incidence of falls among the elderly population significantly impacts their quality of life. Accurate identification of individuals at high risk for falls and the implementation of timely interventions are crucial. Conventional clinical assessment methods can be resource-intensive. Our research aims to effectively address this issue by leveraging sensor-based technology. In our experiments, participants only needed to complete a single TUG task, typically taking less than one minute. This streamlined protocol maximizes efficiency, minimizing time and resource expenditure. We integrated the test with various economically priced and reusable sensor technologies, including IMU and depth cameras, to extract multiple parameters potentially related to fall risk. IMU and depth camera technologies allow for the extraction of different types of features, demonstrating a complementary role in assessing fall risk. Some of these parameters, such as the usage of walking assistance, maximum angular velocity around the yaw axis during turn-to-sit, and step length, exhibit robust discriminatory abilities in identifying high-fall-risk individuals. We provided physiological explanations for their significance, offering crucial insights from a physiological perspective for identifying individuals at high risk of falls.

### Electronic supplementary material

Below is the link to the electronic supplementary material.

**Additional file 1**: *t* test or Mann–Whitney *U* test for all parameters

## Data Availability

The datasets generated and analyzed during the current study are not publicly available due to privacy and ethical concerns but are available from the corresponding author upon reasonable request.
